# The ScreeLing: Detecting Semantic, Phonological, and Syntactic Deficits in the Clinical Subtypes of Frontotemporal and Alzheimer’s Dementia

**DOI:** 10.1177/10731911231154512

**Published:** 2023-02-17

**Authors:** Lize C. Jiskoot, Jackie M. Poos, Kristof van Boven, Liset de Boer, Lucia A. A. Giannini, Djaina D. Satoer, Evy G. Visch-Brink, Judy van Hemmen, Sanne Franzen, Yolande A. L. Pijnenburg, Esther van den Berg, Harro Seelaar

**Affiliations:** 1 Erasmus University Medical Center, Rotterdam, the Netherlands; 2 University College London, UK; 3 Amsterdam University Medical Center, the Netherlands

**Keywords:** linguistic test, frontotemporal dementia, semantics, phonology, syntax, primary progressive aphasia

## Abstract

The ScreeLing is a screening instrument developed to assess post-stroke aphasia, via the linguistic levels Syntax, Phonology, and Semantics. It could also be a useful test for the clinical subtypes of frontotemporal dementia (FTD) and Alzheimer’s dementia (AD), as specific and often selective disorders are expected. Its ability to differentiate between the clinical subtypes of FTD and AD is, however, still unknown. We investigated differences in ScreeLing total and subscores, linguistic-level disorders’ relationship with disease severity, and classification abilities, in patients with behavioral variant FTD (bvFTD; *n* = 46), patients with primary progressive aphasia (PPA; *n* = 105) (semantic variant primary progressive aphasia [svPPA], non-fluent variant primary progressive aphasia [nfvPPA], and logopenic variant primary progressive aphasia [lvPPA], AD [*n* = 20] and controls [*n* = 35]). We examined group differences in ScreeLing total and subscores, and one-, two- or three-level linguistic disorders using one-way analyses of covariance (ANCOVAs) or Quade’s rank ANCOVA. We used frequency analyses to obtain the occurrence of the linguistic-level disorders. We determined sensitivity and specificity by the area under the curve by receiver-operating characteristics analyses to investigate classification abilities. The total score was lower in patients (bvFTD: 63.8 ± 8.5, svPPA: 58.8 ± 11.3, nfvPPA: 63.5 ± 8.4, lvPPA: 61.7 ± 6.6, AD: 63.8 ± 5.5) than controls (71.3 ± 1.0) (*p* < .001). Syntax subscores were lower in svPPA (19.4 ± 4.6; *p* < .001) and lvPPA (20.3 ± 3.2; *p* = .002) than controls (23.8 ± 0.4). Phonology subscores were lower in lvPPA (19.8 ± 2.6) than bvFTD (21.7 ± 2.8) (*p* = .010). Semantics subscores were lowest in svPPA (17.8 ± 5.0; *p* < .002). A selective phonological disorder was most prevalent in lvPPA (34.9%). The higher the disease severity, the more linguistic-level disorders. The optimal cutoff for the total score was 70, and 23 for all three subscores. Good classification abilities were found for the Semantics (svPPA vs. bvFTD), Phonology (lvPPA vs. svPPA), and Syntax (nfvPPA vs. lvPPA) subscores. This easy to administer test gives information about language processing with the potential to improve differential diagnosis in memory clinics and in the future potentially also clinical trial planning.

Frontotemporal dementia (FTD) is a clinically and pathologically heterogeneous type of early-onset dementia, typically characterized by atrophy of the frontal and/or temporal lobes (Olney et al., 2020). The most common clinical subtype is behavioral variant FTD (bvFTD). bvFTD is characterized by marked changes in personality and behavior, such as disinhibition, apathy, loss of empathy, ritualistic, and obsessive–compulsive behaviors, and changes in eating and diet (Rascovsky et al., 2011). Another subtype of FTD is primary progressive aphasia (PPA), as progressive problems in speech and language are the most prominent clinical features. Language and speech disturbances are an integral part of the FTD disease spectrum, both in bvFTD and the three clinical subtypes of PPA—that is, semantic variant primary progressive aphasia (svPPA), non-fluent variant primary progressive aphasia (nfvPPA), and logopenic variant primary progressive aphasia (lvPPA) (Bruni, 2010). Impairments in bvFTD include disorders in, for example, speech production, word retrieval, object naming, word and sentence comprehension, reading, and spelling (Hardy et al., 2016; Paternicó et al., 2016). Core deficits in PPA include impaired confrontation naming and single-word comprehension in patients with svPPA, agrammatism, and apraxia of speech in patients with nfvPPA, and impaired single-word retrieval in spontaneous speech, and naming and impaired repetition of longer phrases and sentences in patients with lvPPA (Gorno-Tempini et al., 2011). svPPA and nfvPPA are often associated with FTD pathology, while more than 50% of lvPPA cases have Alzheimer’s dementia (AD) pathology (Giannini et al., 2017). The clinical overlap between the FTD spectrum disorders, and other neurodegenerative diseases, such as AD, poses an important challenge for early (differential) diagnosis, clinical counseling and planning patient, and caregiver management (Musa et al., 2020). Moreover, early and precise patient stratification is becoming increasingly important with upcoming disease-modifying treatments (Marshall et al., 2018).

Comprehensive language instruments to distinguish between patients with PPA have been developed in the past, such as the Boston Diagnostic Aphasia Examination (BDAE) or the Western Aphasia Battery (WAB). Administration and interpretation of such instruments is, however, time-consuming (Patel et al., 2022). Briefer screening instruments, such as the Mini-Linguistic State Examination (MLSE; Patel et al., 2022), have become available, but are not available or validated in the Dutch language, or cannot reliably disentangle PPA from non-PPA patients (e.g., Sydney Language Battery NL; Janssen et al., 2022). The ScreeLing (“Screening Linguïstiek” in Dutch, or translated to English, “Screening Linguistics”) is a screening instrument for diagnosing aphasia via the linguistic levels Syntax, Phonology, and Semantics. Originally developed as an instrument for post-stroke aphasia (Doesborgh et al., 2003; Visch-Brink et al., 2010), a number of studies have so far been conducted into the occurrence, prognosis, and recovery of linguistic-level deficits as measured by the ScreeLing. For instance, deficits in all three linguistic levels were found in approximately 39% of 141 acute post-stroke patients (the lowest scores for the Phonology subtest) and more severe aphasia (as measured by the Token Test) in patients with deficits in all three linguistic levels (El Hachioui et al., 2012). Also, a systematic review on the availability of screening tests for the differentiation between aphasic and non-aphasic stroke patients and its psychometric properties identified the ScreeLing as one of the only tests having good diagnostic properties (e.g., 86% sensitivity and 96% specificity; El Hachioui et al., 2017). The ScreeLing could also be a useful test for PPA as specific and often selective disorders are expected in svPPA (a semantic disorder), nfvPPA (a syntactic disorder), and lvPPA (a phonological disorder) (Visch-Brink et al., 2010). To date, separate norms for PPA and other neurodegenerative disorders are, however, not available (Visch-Brink et al., 2010). Using the ScreeLing in FTD and AD spectrum disorders could not only provide us more insight into the nature and occurrence of linguistic-level disorders in the most prevalent dementia disorders, but also help differential diagnosis between clinical subtypes as this test taps into the specific disorders (or combination of disorders) found in FTD and AD spectrum disorders.

The aim of the present study was therefore to investigate (a) group differences in the ScreeLing total, Syntax, Phonology, and Semantics scores; (b) the nature and extent of linguistic-level disorders, and the relationship with disease severity; and (c) the classification abilities of the ScreeLing, in patients with bvFTD, PPA (svPPA, nfvPPA, and lvPPA), AD, and controls.

## Method

### Participants

In this retrospective study, we included data from 171 patients with dementia via the outpatient memory clinic of the Erasmus MC University Medical Center, Rotterdam, the Netherlands. Patients were recruited between April 2010 and June 2022. We included 46 patients with bvFTD, 32 patients with svPPA, 30 patients with nfvPPA, 43 patients with lvPPA, and 20 patients with AD. Twenty patients with bvFTD and three patients with nfvPPA were carriers of a pathogenic FTD mutation (*C9orf72, GRN, MAPT*, or *TARDBP*), all other patients were sporadic cases. Clinical diagnoses were made in multidisciplinary consensus meetings, using established diagnostic criteria for probable bvFTD (Rascovsky et al., 2011), PPA (Gorno-Tempini et al., 2011), and AD (McKhann et al., 2011), using all available clinical information (e.g., patient- and informant-based information, neuropsychological tests, magnetic resonance [MR] imaging of the brain, fluid biomarkers). Furthermore, we enrolled 35 healthy control participants from the FTD Risk Cohort (FTD-RisC), in which first-degree family members of patients with genetic FTD are followed longitudinally (Dopper et al., 2014). Inclusion criteria for this control group were being a non-carrier of an FTD mutation (confirmed by DNA genotyping) and having no test score ≥1.5 *SD* below age-, sex-, and education-specific means on neuropsychological assessment.

### Standard Protocol Approvals, Registrations, and Patient Consents

All patients with dementia from the outpatient clinic of the Erasmus MC University Medical Center were part of a local biobank study, for which they provided written informed consent for the use of their anonymized medical and clinical data for research purposes. Participants of the FTD-RisC study provided written informed consent for the use of their anonymized research data. The Erasmus MC University Medical Center ethics committee gave approval for both the local biobank (MEC-2016-069) and the FTD-RisC study (MEC-2009-409).

### Procedure

The ScreeLing was administered as part of the neuropsychological assessment performed during the memory clinic work-up (patients) or study visit (controls). The Mini-Mental State Examination (MMSE; Folstein et al., 1975) and Frontal Assessment Battery (FAB; Dubois et al., 2000) were administered as measures of global cognitive and frontal-executive functioning, respectively. The global score from the Clinical Dementia Rating (CDR) scale (Morris, 1993) was used as a measure of disease severity in patients with AD, whereas patients with FTD (bvFTD and PPA) and controls were assessed with respect to functional changes in behavior, neuropsychiatric symptoms, cognition, and language using the CDR plus National Alzheimer’s Coordinating Center (NACC) Frontotemporal Lobar Degeneration (FTLD; Miyagawa et al., 2020). Other neuropsychological tests administered to patients and controls were the 60-item Boston Naming Test (BNT; Kaplan et al., 1978), verbal Semantic Association Test (SAT; Visch-Brink et al., 2005), semantic and letter fluency (Thurstone & Thurstone, 1962), Visual Association Test (VAT) short form (Lindeboom et al., 2002), and Trail Making Test (TMT) parts A and B (Battery Army Individual Test, 1994).

### ScreeLing

The ScreeLing investigates the performance on the three main linguistic levels Syntax (sentence structure), Phonology (patterns of speech sounds), and Semantics (meaning) (Doesborgh et al., 2003; Visch-Brink et al., 2010). Every subtest consists of 24 items (maximum score) across four task groups with a maximum total score of 72. See Appendix 1 for a more detailed description of the test and its subtests. The Syntax subtest consists of sentence-picture matching (eight items), who/what/where questions (four items), identifying syntactic incorrect sentences (six items), and sentence completion with function words (six items). The Phonology subtest consists of: repetition multisyllabic words and phrases (six items), reading aloud multisyllabic words and phrases (six items), judgment equal/unequal word pairs (six items), and matching phonemes with letters (six items). The Semantics subtest consists of: word–picture matching (six items), identifying semantically anomalous sentences (six items), verbal semantic association (six items), and categorizing lexical items (odd-word out) (six items). To avoid deficits in the auditory or reading input channel, all items, except repetition and reading aloud, are presented in written words, which may be read aloud by the examiner, if necessary. Test duration is between 20 and 45 minutes, with an average of 30 minutes. Reliability and validity of the ScreeLing was evaluated in patients with aphasia due to stroke (*n* = 147) and control subjects (*n* = 138) (Visch-Brink et al., 2010). There was a high internal consistency for the total ScreeLing, the subscales and their components (Cronbach’s alpha and Kuder–Richardson Formula 20 coefficient was 0.95 for the total ScreeLing, 0.93–0.95 for the subscales and 0.71–0.92 for the components). The average-corrected item total correlation was 0.79 for the total ScreeLing, 0.57 to 0.64 for the subscales and 0.42 to 0.77 for the components. The test–retest reliability was high (Spearman’s rho and Pearson’s correlations between measurements was between 0.86 and 0.96, *p <* .001). The internal and external structures were considered good, with moderate to high Pearson’s correlations between the ScreeLing total and subscores (*r* = 0.78–0.97) and between ScreeLing total and subscores and the 36-item Token Test (*r* = 0.69–0.91). Receiver-operating characteristic (ROC) analyses on the ScreeLing total showed a 94% sensitivity and 81% specificity between patients with aphasia due to stroke and controls, with a cutoff of 68 (i.e., ≥ 68 is considered normal). The cutoff for all three subscores was determined at 22 (sensitivity 91%–94% and specificity 56%–83%).

### Statistical Analysis

We performed statistical analyses using SPSS Statistics 28.0.1.0 (IBM Corp., Armonk, NY, USA) and GraphPad Prism 7 (La Jolla, CA, USA). Alpha was set at 0.05 across all comparisons (two-tailed). We compared continuous demographic data between groups using one-way analysis of variance (ANOVA) for normally distributed data (with Bonferroni post hoc tests), or Kruskal–Wallis tests for non-normally distributed data (with Mann–Whitney *U* post hoc tests). To explore construct validity, we investigated associations between the ScreeLing total and subscores, and other language (BNT, SAT verbal, semantic, and letter fluency) and cognitive tests (VAT, TMT A, and B) using partial correlations (Spearman’s rank or Pearson’s correlation, where appropriate). Age, sex, and education levels were added as covariates. We analyzed between-group differences in sex distribution with Pearson’s χ^2^ tests. We examined group differences in the ScreeLing total score and subscores using one-way ANCOVAs for normally distributed data, or Quade’s rank ANCOVA for non-normally distributed data. Age, sex, and education levels were added as covariates. We used frequency analyses to obtain the occurrence of the linguistic-level disorders in patients. We investigated associations between the ScreeLing total and subscores and measures of disease severity (i.e., CDR [plus NACC FTLD], MMSE, and FAB) using partial correlations (Spearman’s rank or Pearson’s correlation, where appropriate). Age, sex, and education levels were added as covariates. We examined differences in the ScreeLing total and subscores and one-, two- or three-level linguistic disorders using one-way ANCOVAs when data were normally distributed, or Quade’s rank ANCOVA when data were non-normally distributed. Age, sex, and education levels were added as covariates. We determined sensitivity and specificity by the area under the curve (AUC) by ROC analyses to investigate the classification abilities of the ScreeLing total and subscores. An AUC > 0.80 is considered to have excellent discrimination abilities (Hosmer et al., 2013). Optimal cutoff levels were given by the highest Youden’s index (Youden, 1950). All models were corrected for multiple comparisons (Bonferroni).

## Results

### Demographics and Clinical Data

Demographic and clinical data are shown in Table 1. Controls were significantly younger than the patient groups (all *p* < .001), and patients with bvFTD were significantly younger than patients with nfvPPA (*U* = 441.5, *p* = .008), lvPPA (*U* = 431.5, *p* < .001), and AD (*U* = 204.0, *p* < .001]. The CDR (plus NACC FTLD) was significantly lower in controls than in the patients groups (all *p* < .001), but there were no significant differences between patient groups (all *p* > .05). MMSE scores were significantly higher in controls than in patients with svPPA (*U* = 65.0, *p* < .001), lvPPA (*U* = 53.5, *p* < .001), and AD (*U* = 6.0, *p* < .001). Patients with bvFTD had higher MMSE scores than patients with lvPPA (*U* = 429.0, *p* < .001), and AD (*U* = 186.0, *p* < .001), and patients with nfvPPA had higher MMSE scores than patients with lvPPA (*U* = 230.5, *p* < .001). Controls had higher FAB scores than patients with bvFTD (*p* = .003), nfvPPA (*p* = .010), and lvPPA (*p* < .001). There were no group differences in sex, *X*(5) = 5.574, *p* = .350, and education levels, *F*(5, 187) = 0.526, *p* = .756.

**Table 1:** Demographic and Clinical Data Per Subgroup

Variable	bvFTD	svPPA	nfvPPA	lvPPA	AD	Controls	Difference
**Number of participants**	46	32	30	43	20	35	n/a
**Age, y**	59.5 (10.2)	63.2 (7.4)	66.0 (9.0)	69.4 (7.5)	69.3 (7.9)	51.7 (8.5)	con < bvFTD < nfvPPA = lvPPA = AD
**Sex**, *F* **(%)**	20 (43.5)	14 (43.8)	20 (66.7)	24 (55.8)	9 (45.0)	17 (48.6)	–
**Education level** ^a^	5.0 (1.1)	5.1 (1.4)	5.0 (1.2)	4.9 (1.3)	4.7 (1.0)	5.4 (0.8)	–
**CDR** ^©^ **(plus NACC FTLD), range** ^b^	1.1 (0.6) (0.5–2.0)	0.7 (0.3) (0.5–1.0)	0.8 (0.6) (0–2.0)	0.7 (0.3) (0.5–1.0)	1.1 (0.6) (0.5–2.0)	0 (–)	con < bvFTD = svPPA = nfvPPA = lvPPA = AD
**Neuropsychological assessment**							
**MMSE (max. 30)**	26.2 (3.7)	23.6 (6.1)	26.4 (3.8)	22.2 (5.5)	22.3 (4.5)	29.2 (0.9)	AD = svPPA = lvPPA < bvFTD = nfvPPA = con
**FAB (max. 18)**	13.0 (3.7)	14.1 (3.7)	12.9 (4.0)	12.7 (3.3)	12.7 (3.3)	16.9 (1.4)	bvFTD = nfvPPA = lvPPA < con
**ScreeLing—total score (max. 72)**	63.8 (8.5)	58.8 (11.3)	63.5 (8.4)	61.7 (6.6)	63.8 (5.5)	71.3 (1.0)	bvFTD = svPPA = nfvPPA = lvPPA = AD < con
**ScreeLing—Syntax (max. 24)**	21.2 (3.4)	19.4 (4.6)	21.0 (3.9)	20.3 (3.2)	21.2 (2.6)	23.8 (0.4)	svPPA = lvPPA < con
**ScreeLing—Phonology (max. 24)**	21.7 (2.8)	21.6 (2.7)	20.5 (3.2)	19.8 (2.6)	21.1 (2.6)	23.8 (0.3)	lvPPA < bvFTD = svPPA = nfvPPA = AD < con
**ScreeLing—Semantics (max. 24)**	21.0 (3.3)	17.8 (5.0)	22.0 (2.9)	21.6 (2.2)	21.4 (2.2)	23.7 (0.6)	svPPA < bvFTD = nfvPPA = lvPPA < AD = con
**BNT (max. 60)**	39.7 (13.6)	14.8 (10.9)	49.0 (10.7)	33.8 (13.1)	35.6 (14.1)	54.3 (3.8)	svPPA < lvPPA < bvFTD = AD < nfvPPA = con
**SAT verbal (max. 30)**	24.7 (4.0)	19.8 (6.9)	26.8 (2.3)	24.9 (2.9)	25.7 (4.5)	28.1 (1.0)	svPPA < bvFTD = nfvPPA = AD < lvPPA < con
**Semantic fluency (animals)**	14.1 (5.1)	9.4 (5.4)	14.8 (6.0)	11.3 (6.1)	11.3 (4.4)	26.9 (6.8)	svPPA < bvFTD = nfvPPA = AD = lvPPA < con
**Letter fluency (total three letters)**	21.5 (11.6)	24.3 (11.9)	17.6 (10.7)	16.6 (8.8)	15.1 (11.6)	39.7 (13.5)	bvFTD = svPPA = nfvPPA = lvPPA = AD < con
**VAT (max. 12)**	10.3 (2.9)	8.5 (2.6)	11.0 (1.6)	8.6 (3.8)	7.0 (3.2)	11.9 (0.2)	AD = svPPA < lvPPA < nfvPPA = bvFTD = con
**TMT A, seconds**	65.7 (47.5)	53.9 (33.7)	68.6 (49.0)	100.6 (64.3)	116.8 (88.8)	28.2 (8.0)	con = nfvPPA = svPPA = bvFTD < lvPPA = AD
**TMT B, seconds**	185.2 (98.5)	140.0 (88.5)	190.9 (81.6)	232.3 (84.0)	276.1 (54.0)	59.5 (11.2)	con < nfvPPA = svPPA = bvFTD < lvPPA = AD

*Note.* Values indicate *M* (*SD*) or *n* (%). bvFTD = behavioral variant frontotemporal dementia; svPPA = semantic variant primary progressive aphasia; nfvPPA = non-fluent variant primary progressive aphasia; lvPPA = logopenic variant primary progressive aphasia; AD = Alzheimer’s dementia; con = controls; *F* = female; MMSE = Mini-Mental State Examination; FAB = Frontal Assessment Battery; CDR = Clinical Dementia Rating; NACC = National Alzheimer’s Coordinating Center; FTLD = frontotemporal lobar degeneration; BNT = Boston Naming Test; SAT = Semantic Association Test; VAT = Visual Association Test; TMT = Trail Making Test.

a Dutch educational system categorized into levels from 1 = less than 6 years of primary education to 7 = academic schooling (Duits et al., 2014). ^b^ The CDR-weighted score was used for patients with AD, whereas the CDR^©^ plus NACC FTLD-weighted score was used for patients with bvFTD, PPA, and controls.

### Construct Validity

Partial correlations between the ScreeLing total and subtest scores, and other language (BNT, SAT verbal, semantic, and letter fluency) and other cognitive tests (VAT, TMT A, and B) are shown in Appendix 2. Medium to strong correlations (*r* between 0.3 and 0.6) were found between the ScreeLing total and Semantics subscore and all other language measures. Moreover, the Syntax subscore correlated with SAT verbal, while medium correlations were found between the Phonology and Syntax subtests, and letter fluency. The ScreeLing total and subtests did not correlate with the other cognitive tests.

### Group Differences in the ScreeLing

**Figure 1.**
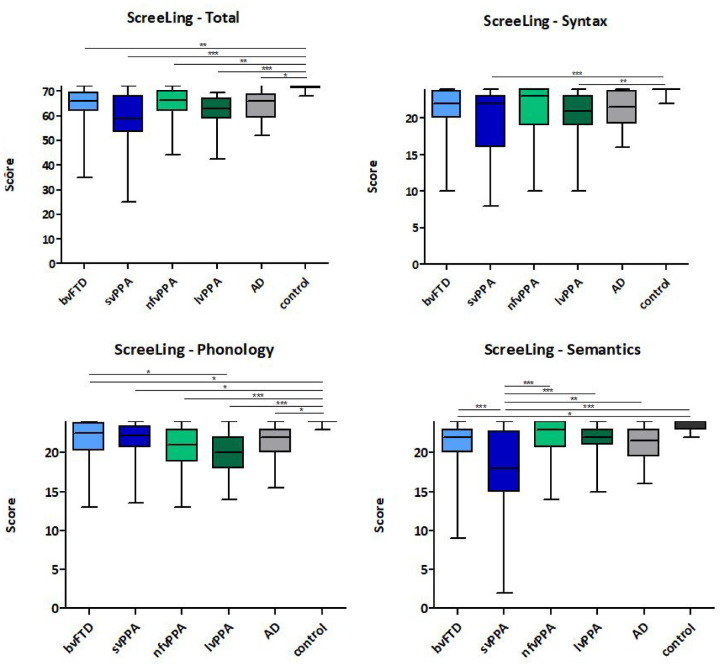
Boxplots Showing Group Differences in ScreeLing Total, Syntax, Phonology and Semantics Scores Between Patients and Controls. *Note*. Values indicate median and the box extends from the 25th to 75th percentiles, the whiskers give minimum and maximum values. bvFTD = behavioral variant frontotemporal dementia; svPPA = semantic variant primary progressive aphasia; nfvPPA = non-fluent variant primary progressive aphasia; lvPPA = logopenic variant primary progressive aphasia; AD = Alzheimer’s dementia. *p < .05. **p < .010. ***p < .001.

There were significant differences in ScreeLing total score between groups, *F*(5, 184) = 6.781, *p* < .001 (Table 1, Figure 1). All patient groups had lower ScreeLing total scores than controls (all *p* < .05), but there were no significant differences between patient groups (all *p* > .05). With respect to the subscores, group differences were found for Syntax, *F*(5, 185) = 4.782, *p* < .001; Phonology, *F*(5, 186) = 7.588, *p* < .001; and Semantics *F*(5, 185) = 10.546, *p* < .001. The Syntax subscores were lower in patients with svPPA (*p* < .001) and lvPPA (*p* = .002) than in controls, and trends were found in patients with bvFTD (*p* = .058) and nfvPPA (*p* = .050) in comparison with controls. No differences were found between patient groups. The Phonology subscore was lower in all patient groups in comparison with controls (all *p* < .05), and was also significantly lower in patients with lvPPA than in patients with bvFTD (*p* = .010). The Semantics subscore was lower in patients with bvFTD (*p* = .028) and svPPA (*p* < .001) than in controls. Moreover, patients with svPPA had significantly lower subscores than all other patient groups (all *p* < .002). Patients with AD did not significantly differ from controls or other dementia patients (all *p* > .05).

### Linguistic-Level Disorders and Relationship With Disease Severity

**Figure 2.**
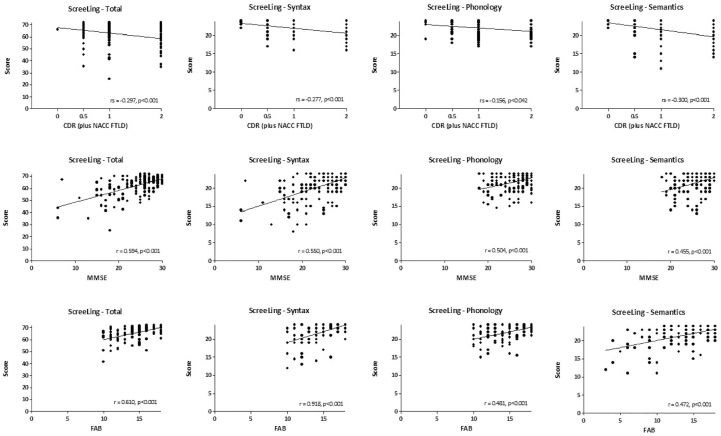
Partial Correlations Between the ScreeLing Total, Syntax, Phonology and Semantics and CDR (Plus NACC FTLD) (Top Row), MMSE (Middle Row) and FAB (Bottom Row). *Note.* Displayed are Spearman’s rank (CDR [plus NACC FTLD]) and Pearson’s (MMSE and FAB) correlations and *p*-values. Age, sex, and education levels were added as covariates. CDR = Clinical Dementia Rating; NACC = National Alzheimer’s Coordinating Center; FTLD = frontotemporal lobar degeneration; MMSE = Mini-Mental State Examination; FAB = Frontal Assessment Battery.

Using the cutoff score of 68 for the ScreeLing total score (El Hachioui et al., 2012; Visch-Brink et al., 2010), the largest proportion of patients (~70%) showed a score in the disordered range. Most scores in this range were found in patients with lvPPA and AD, followed by patients with svPPA, nfvPPA, and bvFTD (Table 2). Only one control subject had a total score below the cutoff. Selective linguistic-level disorders occurred in about a third of patients, that is, they scored in the disordered range on one subtest, while performing normal on the other two linguistic levels (Table 2). A selective phonological disorder occurred most frequently. The selective phonological disorder was primarily found in patients with lvPPA, followed by patients with bvFTD and nfvPPA. A selective syntactic or a selective semantic disorder was relatively rare (Table 2). Among the combined disorders of two linguistic levels, the most frequent was the combination of a phonological and syntactic deficit, mostly occurring in patients with lvPPA. A combined disorder in the other linguistic levels was rare (between 3.0% and 4.7% of patients) (Table 2). A three-level disorder was found in about a fifth, the most common diagnoses being svPPA, lvPPA, and bvFTD (Table 2). Just over 20% of patients did not have a disorder on any of the linguistic levels (Table 2). A higher disease severity, as expressed by higher CDR (plus NACC FTLD), and lower MMSE and FAB scores, correlated significantly with lower ScreeLing total, Syntax, Phonology, and Semantics scores (Figure 2). There was an overall difference in disease severity between patients with a selective linguistic-level disorder, a combined disorder of two linguistic levels, and a three-level deficit, MMSE H(3) = 60.951, *p* < .001; FAB H(3) = 52.959, *p* < .001; CDR (plus NACC FTLD) H(3) = 23.542, *p* < .001. Patients with a three-level disorder had the lowest MMSE and FAB, and highest CDR (plus NACC FTLD) scores, followed by patients with a combined disorder of two linguistic levels, then patients with a selective linguistic-level disorder and finally patients without a linguistic disorder (Appendix 3).

**Table 2:** Number (%) of Patients With a One-, Two-, or Three-Level Linguistic Disorder on the ScreeLing

Type of disorder	All patients	bvFTD	svPPA	nfvPPA	lvPPA	AD
**Number of participants**	171	46	32	30	43	20
**Total score**	118 (69.8)	26 (56.5)	22 (68.8)	18 (60.0)	37 (86.0)	15 (75.0)
**No disorder**	37 (21.9)	15 (32.6)	7 (21.9)	9 (30.0)	2 (4.7)	4 (20.0)
**One-level disorder**						
**Selective syntactic disorder**	11 (6.5)	3 (6.5)	1 (3.1)	2 (6.7)	2 (4.7)	3 (15.0)
**Selective phonological disorder**	41 (24.3)	9 (19.6)	4 (12.5)	8 (26.7)	15 (34.9)	5 (25.0)
**Selective semantic disorder**	8 (4.7)	2 (4.3)	3 (9.4)	1 (3.3)	1 (2.3)	1 (5.0)
**Two-level disorder**						
**Syntactic and phonological disorder**	24 (14.2)	4 (8.7)	0 (0)	4 (13.3)	14 (32.6)	2 (10.0)
**Syntactic and semantic disorder**	5 (3.0)	2 (4.3)	2 (6.3)	0 (0)	0 (0)	1 (5.0)
**Phonological and semantic disorder**	5 (3.0)	1 (2.2)	3 (9.4)	0 (0)	0 (0)	1 (5.0)
**Three-level disorder**						
**Semantic, phonological, and syntactic disorder**	38 (22.5)	8 (17.4)	12 (37.5)	6 (20.0)	9 (20.9)	3 (15.0)

*Note.* Values indicate *n* (%). Missing data in two patients with bvFTD: only Phonology data were available (both bvFTD). bvFTD = behavioral variant frontotemporal dementia; svPPA = semantic variant primary progressive aphasia; nfvPPA = non-fluent variant primary progressive aphasia; lvPPA = logopenic variant primary progressive aphasia; AD = Alzheimer’s dementia.

### Classification Abilities of the ScreeLing

The classification abilities of the total ScreeLing, and Syntax, Phonology, and Semantics subscores can be found in Table 3. ROC analyses showed that the ScreeLing total and subscores discriminate significantly between the total group of dementia patients and controls (sensitivity between 58.3 and 92.3%, specificity between 86.4% and 90.9%; AUC > 0.80, i.e., excellent to outstanding discrimination). The optimal cutoff score for the total ScreeLing was 70, that is, participants scoring less than 70 were classified as patient with dementia. The optimal cutoff score for all three subscores was 23. The ScreeLing also discriminated accurately between the specific dementia groups and controls, though best discriminative abilities were found for the total score, and worst discriminative abilities were found for the Semantics subtest (sensitivity between 36.7% and 68.8%; AUC > 0.60, i.e., poor to excellent discrimination). The optimal cutoffs for the total ScreeLing were 69–70; the optimal cutoffs for the three subscores were 22–23. No score accurately discriminated between patients with bvFTD and patients with AD (*p* > .05). Patients with svPPA could be distinguished from patients with bvFTD and patients with nfvPPA based on their Semantics subscore, with a score lower than 20 being indicative for svPPA. The scores on the Phonology and Semantics subtests discriminated accurately between patients with svPPA and lvPPA, with scores, respectively, lower than 18 and higher than 21 being indicative for lvPPA. Finally, patients with nfvPPA could be distinguished from patients with lvPPA based on their Syntax subscore, with a score lower than 23 being indicative for nfvPPA.

**Table 3:** Classification Abilities of the ScreeLing Total and Subscores

Group	AUC	[95% CI]	*p* value	Optimal cutoff	Sensitivity (%)	Specificity (%)
**Patient vs. control**						
Total	.95	[.92 – .99]	< .001	70	92.3	90.9
Syntax	.86	[.80 – .92]	< .001	23	79.2	86.4
Phonology	.88	[.83 – .93]	< .001	23	81.5	86.4
Semantics	.81	[.74 – .88]	< .001	23	58.3	90.9
**bvFTD vs. control**						
Total	.93	[.87 – 1.00]	< .001	70	90.9	91.0
Syntax	.83	[.73 – .93]	< .001	23	52.3	95.5
Phonology	.81	[.71 – .91]	< .001	23	50.0	100
Semantics	.84	[.75 – .94]	< .001	23	56.8	90.9
**svPPA vs. control**						
Total	.94	[.87 – 1.00]	< .001	69	84.4	95.5
Syntax	.86	[.76 – .96]	< .001	23	78.1	86.4
Phonology	.85	[.75 – .95]	< .001	23	75.0	86.4
Semantics	.89	[.80 – .98]	< .001	22	68.8	100
**nfvPPA vs. control**						
Total	.93	[.86 – 1.00]	< .001	70	90.0	90.9
Syntax	.80	[.67 – .92]	< .001	23	70.0	86.4
Phonology	.89	[.52 – .81]	< .001	23	86.7	86.4
Semantics	.67	[.52 – .81]	.039	23	36.7	90.9
**lvPPA vs. control**						
Total	.99	[.97 – 1.00]	< .001	70	100	95.5
Syntax	.94	[.88, 1.00]	< .001	23	93.0	86.4
Phonology	.98	[.95, 1.00]	< .001	22	86.0	100
Semantics	.80	[.70 – .91]	< .001	23	60.5	90.9
**AD vs. control**						
Total	.97	[.92 – 1.00]	< .001	70	94.7	90.9
Syntax	.83	[.70 – .97]	< .001	23	73.7	86.4
Phonology	.87	[.75 – .99]	< .001	23	78.9	86.4
Semantics	.82	[.69 – .96]	< .001	23	63.0	90.9
**bvFTD vs. AD**						
Total	.56	[.41 – .71]	.449	—	—	—
Syntax	.53	[.37 – .69]	.714	—	—	—
Phonology	.57	[.41 – .72]	.410	—	—	—
Semantics	.50	[.35 – .66]	.964	—	—	—
**svPPA vs. nfvPPA**						
Total	.61	[.46 – .75]	.149	—	—	—
Syntax	.61	[.47 – .75]	.139	—	—	—
Phonology	.40	[.26 – .55]	.190	—	—	—
Semantics	.77	[.65 – .89]	< .001	20	59.4	86.7
**svPPA vs. lvPPA**						
Total	.54	[.39 – .68]	.574	—	—	—
Syntax	.51	[.36 – .65]	.944	—	—	—
Phonology	.73	[.61 – .86]	.001	21	75.0	67.4
Semantics	.73	[.60 – .85]	.001	18	50.0	95.3
**nfvPPA vs. lvPPA**						
Total	.63	[.49 – .77]	.063	—	—	—
Syntax	.64	[.50 – .78]	.042	23	69.8	60.0
Phonology	.61	[.47 – .75]	.118	—	—	—
Semantics	.60	[.46 – .74]	.140	—	—	—
**svPPA vs. bvFTD**						
Total	.63	[.50 – .76]	.054	—	—	—
Syntax	.60	[.47 – .73]	.148	—	—	—
Phonology	.52	[.39 – .65]	.744	—	—	—
Semantics	.69	[.56 – .82]	.005	20	59.4	79.5

*Note.* AUC = area under the curve; CI = confidence interval; bvFTD = behavioral variant frontotemporal dementia; svPPA = semantic variant primary progressive aphasia; nfvPPA = non-fluent variant primary progressive aphasia; lvPPA = logopenic variant primary progressive aphasia; AD = Alzheimer’s dementia.

## Discussion

This study investigated group differences, the nature and extent of linguistic-level disorders and the relationship with disease severity, and the classification abilities of the ScreeLing in patients with bvFTD, PPA, AD, and cognitively healthy controls. We also explored construct validity. The ScreeLing total score was overall lower in patients than in controls, as well as the Syntax, Phonology, and Semantics subscores in patients with svPPA and lvPPA, lvPPA, and svPPA, respectively. Patients with AD did not significantly differ from controls or other dementia patients. Most scores in the disordered range were found in patients with lvPPA and AD. A selective phonological disorder and combined phonological-syntactic disorders were primarily found in patients with lvPPA, while a three-level disorder occurred most often in patients with svPPA. The higher the disease severity, the more linguistic-level disorders. The optimal cutoff for the total score was 70, while the optimal cutoff score for all three subscores was 23. Patients with svPPA could be distinguished from patients with bvFTD and nfvPPA based on their Semantics subscore, patients with lvPPA from patients with svPPA based on their Phonology subscore, and finally, patients with nfvPPA could be distinguished from patients with lvPPA based on their Syntax subscore. No score accurately discriminated between patients with bvFTD and patients with AD.

We found group differences for the ScreeLing Syntax, Phonology, *and* Semantics subtests, reflecting different disorders at one or more linguistic levels. The lowest score was found for the Semantics subscore in patients with svPPA. This is not a surprising finding, given that deficits in semantic memory and processing form the core problem in svPPA (Landin-Romero et al., 2016), and are thus well-captured by the four components of the subtest (i.e., word-image matching, determining whether a sentence is semantically correct, word association, categorizing). Moreover, patients with bvFTD had significantly lower Semantics subscores than controls, in line with previous research showing semantic deficits in bvFTD (Hardy et al., 2016). Patients with svPPA also attained low scores on the Syntax subtest. This is unexpected, as patients are thought to retain not only phonological but also syntactic abilities (Kertesz et al., 2010). It can be assumed that the Syntax scores are affected by the profound deficits in semantic knowledge (i.e., lexical comprehension; Charles et al., 2014) and not due to a *pure* syntactic disorder. This hypothesis is supported by high partial correlations between the SAT verbal, a test for semantic knowledge, and all aspects (total and all three subscores) of the ScreeLing. In lvPPA, the lower Syntax scores might be the result of impairment of a large-scale, frontotemporal sentence processing network (Charles et al., 2014). The Phonology subscore was lower in all patient groups in comparison to controls, but specifically in patients with lvPPA. The Phonology subscore thereby seems to capture the core problem in lvPPA, namely, a deficit in the auditory verbal short-term memory—“the phonological loop”—leading to repetition problems in this previously called logopenic/phonological variant of PPA (Gorno-Tempini et al., 2008). Although agrammatism is one of the core features (Gorno-Tempini et al., 2011), only a trend for lower Syntax performance was found in nfvPPA patients. It can be hypothesized that this subtest measures syntactic processing, but not specifically agrammatism or apraxia of speech (both core symptoms) as no verbal output is required. Moreover, the component in which a patient has to determine if a sentence is syntactically correct is most likely too easy, as these sentences are not long and/or grammatically complex enough (e.g., no use of dependent clauses). A trend was found for a lower Syntax score in bvFTD. Grammatical comprehension deficits in this subtype are thought to be not primarily linguistic in nature, but caused by executive dysfunction (e.g., working memory, strategic organization, attention) due to atrophy of the prefrontal areas (Charles et al., 2014).

A phonological disorder was the most common selective linguistic-level disorder, found in about a quarter of patients. In the validation study of the ScreeLing (El Hachioui et al., 2012), a selective phonological disorder also occurred most frequently and coincided with higher spontaneous speech ratings. This suggests that phonological function largely contributes to verbal communication, potentially more than semantic function as was found by other studies (Doesborgh et al., 2002). In line with previous research (El Hachioui et al., 2012), a selective semantic disorder was the least frequent, and also rarely occurred in combination with just one other linguistic-level deficit. As the semantic level tends to be central in language processing, a semantic deficit also affects the performance on the phonological and/or syntactic levels (Patternson & Shewell, 1987). As previously found by El Hachioui et al. (2012), in which the number of linguistic-level disorders was related to the severity of aphasia, we found the number of linguistic-level disorders to be related to disease severity. Interestingly, both in patients with bvFTD and nfvPPA, there was a relatively high percentage of patients not having any linguistic-level disorder. Potentially these patients are in early disease stages in which the language symptoms are still mild (and therefore are not picked up by the ScreeLing) or different than what is currently included in the test (e.g., noun naming is affected early in bvFTD, but not tested in the ScreeLing).

The ScreeLing total score could not classify the dementia subtypes, which suggests that for differential diagnosis the ScreeLing subscores are more useful than the total score. Indeed, a lower Semantics subscore is indicative for svPPA, a lower Phonology subscore but a higher Semantics subscore suggests lvPPA, and a lower Syntax subscore is indicative for nfvPPA. The high sensitivity and specificity to distinguish between bvFTD and svPPA (based on the Semantics subscore) and between nfvPPA and lvPPA (based on the Syntax subscore) are particularly valuable, as differential diagnosis between these disease entities is sometimes difficult in clinical practice. It is interesting that no ScreeLing score accurately discriminated between patients with bvFTD and patients with AD. A potential explanation can be found in the more atypical presentations of AD that are seen in academic outpatient memory clinics such as ours, including patients with the behavioral/dysexecutive variant that shows clinical overlap with bvFTD (Ossenkoppele et al., 2015). The optimal cutoffs for the total ScreeLing were 69–70; the optimal cutoffs for the three subscores were 22–23. These values are somewhat higher than the original cutoffs determined in the validation study of the ScreeLing (El Hachioui et al., 2012). A likely explanation is that patients included in that study were acute stroke patients in which the aphasia severity was higher than in our sample of dementia patients who were being cognitively assessed in a relatively early disease stage (showcased by average CDR (plus NACC FTLD) scores ≤1.1).

A key strength of this study is our large sample of patients, covering the different clinical FTD subtypes. The ScreeLing is a short and easy to administer test that gives information about language processing at the levels of syntax, phonology and semantics. An important advantage of the ScreeLing is that the input—apart from the first component of the Phonology subtask—is given both visually and auditory, therefore, the linguistic level and not the modality (written or spoken language) is the primary point of engagement (Visch-Brink et al., 2010). It could be argued that a study of this type entails a certain degree of circularity, in that the ScreeLing is used in the diagnostic process, and therefore shows group differences and good classification abilities between the FTD spectrum disorders. However, in our multidisciplinary meeting we followed the international consensus criteria for bvFTD (Rascovsky et al., 2011) and PPA (Gorno-Tempini et al., 2011), using all available clinical information—so that, diagnosis did not solely depend on the results of the ScreeLing as part of the larger neuropsychological assessment. Since most clinical diagnoses were not pathologically confirmed, there is a small possibility that patients were misdiagnosed (e.g., patients with behavioral/dysexecutive AD as bvFTD, and bvFTD patients with prominent memory deficits as AD). Currently, we have only included patients with a “pure” PPA variant in our study. A direction for future research entails the inclusion of patients with mixed-types of PPA, as previous research has showed that up to 40% of PPA patients does not fall into one of the three canonical syndromes (Marshall et al., 2018). Moreover, longitudinal analyses as well as analyses in the different genetic subgroups of FTD will provide more information about the progression of language deficits in the FTD disease course and differences in language profile between *GRN, MAPT*, and *C9orf72* mutations, respectively. Finally, unfortunately beyond the scope of the current study, it will be interesting to investigate if the ScreeLing has better differential diagnostic abilities above other language batteries (e.g., BDAE, WAB) or other well-known language test, such as the verbal fluency tests and BNT. A recent systematic review in stroke patients showed that the ScreeLing has one of the best diagnostic properties (in terms of sensitivity/specificity and risk of bias) in comparison with other language screening instruments (El Hachioui et al., 2017).

## Conclusion

Our study investigated group differences, the nature and extent of linguistic-level disorders and the relationship with disease severity, and the classification abilities of the ScreeLing in patients with bvFTD, PPA, AD, and controls. This easy to administer test gives information about language processing at the three main levels of syntax, phonology, and semantics, with the potential to improve clinical practice (differential diagnosis, clinical counseling and planning patient, and caregiver management) and in the future potentially also clinical trial planning (e.g., patient recruitment, stratification, monitoring).

## Appendix 1. The ScreeLing

The test consists of three linguistic components with 24 items across four different tasks:

### Semantics

1. Word–picture matching (six items); six photos of objects, five semantically related foils. A traditional task for semantic processing. Choosing out of semantically related objects requires more of semantic processing than a choice out of unrelated objects. Example: *gorilla*, tiger, elephant, polar bear, wolf, and giraffe.
2. Identifying semantically anomalous sentences (six items); choice correct/incorrect. This task requires recognizing the violation of semantic selection restrictions. The meaning of a word has to be processed in relation to its context. Example: “The ice chose the wrong direction.”
3. Verbal semantic association (six items); choice out of four words, that is, one correct, two distracters semantically related with the target word, and one unrelated distracter. Differentiating between relevant and irrelevant semantic features of a word depending on the required association is necessary. Example: letter: chalk, paint, *pen*, and grass.
4. Odd-word out (six items); choice out of four. The word that does not fit into the same semantic category of three other words has to be selected. Example: violin, *siren*, trumpet, and piano.

### Phonology

1. Repetition of words and phrases (six items); a traditional task for examining phonological disorders in the output route. Phonological complexity is varied according to word length, consonant clusters, identical vowels, phoneme–grapheme correspondence. Example: “monopolie” (*monopoly*); “de excentrieke antiekhandelaar” (*the eccentric antique dealer*).
2. Reading aloud words and phrases (six items); the level of complexity matches the repetition task. Phonological processing may vary depending on the input route. Example: “macaroni” (macaroni), and “de enthousiaste beroepsgoochelaar” (*the enthusiastic professional magician*).
3. Equal/unequal judgment of spoken word pairs (six items); phoneme discrimination, choice yes/no. A well-known task to examine the phonological input route. Example: “straat-staart” (*street-tail*).
4. Matching first phoneme of a spoken word with the grapheme (six items); choice out of three. Phoneme analysis and phoneme-grapheme conversion is required. Example: “boek” (*book*): g, k, and *b*.

### Syntax

1. Sentence-picture matching (eight items); choice out of three or four photographs. The task requires syntactic comprehension, including reversible sentences, subject–verb agreement, reflexive verbs, passive sentences, prepositions, and verb tense. Example: sentence “The man’s hair is being cut by the woman”; three pictures (a) “the man’s hair is being cut by the man,” (b) “the woman’s hair is being cut by the man,” (c) “the man’s hair is being cut by the woman.”
2. Wh-questions (four items); a photographed situation with a “Wh”-question. “Wh”-questions require syntactic processing of the non-canonical sentence construction. Example: “Wie ziet dat hij een taartje pakt?” (*Literally*: “Who sees that he takes a cake?”) The photograph depicts a man and woman talking, while a boy takes a cake. The woman is looking at the boy.
3. Identifying syntactic incorrect sentences (six items); choice correct/incorrect. This task requires processing of word order, subject-verb agreement, auxiliaries, and conjunctions. Example: “Die bloemen is veel te duur” (*Those flowers is far too expensive*).
4. Sentence completion with function words (six items); choice out of four words or phrases. Foils are well-known for addressing syntactic processing: personal pronouns, arguments, prepositions, auxiliaries, and different forms of verb tense or transitive/intransitive verbs. Example: “De jongen geeft zijn vriendin … . . ” (*The boy gives his girlfriend* . . .) Naar de film (*to the movies*), *parfum (perfume)*, wandelen (*hiking*), van de chauffeur (*of the driver*).

**Appendix 2:** Partial Correlations Between the ScreeLing and Other Language and Cognitive Tests

ScreeLing	BNT	SAT verbal	Semantic fluency	Letter fluency	VAT	TMT A	TMT B
**ScreeLing**—**total**	.298*	.487***	.353**	.404***	.087	–.198	–.111
**ScreeLing—Semantics**	.528***	.575***	.434***	.278*	.113	–.126	–.143
**ScreeLing—Phonology**	–.027	.134	.162	.373***	–.040	–.171	–.070
**ScreeLing—Syntax**	.181	.387***	.214	.269*	.114	–.150	–.045

*Note.* Values indicate partial correlation coefficients, corrected for age, sex, and education levels. BNT = Boston Naming Test; SAT = Semantic Association Test; VAT = Visual Association Test; TMT = Trail Making Test.

* *p* < .05. ***p* < .01. ****p* < .001.

**Appendix 3:** Group Differences in Patients With No, One-, Two-, and Three-Level Linguistic Disorders in Disease Severity Scores (CDR^©^ [Plus NACC FTLD], MMSE, and FAB

	No disorders	One-level disorder	Two-level disorder	Three-level disorder	Difference
**CDR** ^©^ **(plus NACC FTLD)** ^a^	0.4 (0.6)	0.7 (0.3)	0.9 (0.4)	1.4 (0.7)	No > 1 = 2 > 3
**MMSE**	28.1 (2.1)	25.4 (4.4)	23.9 (4.4)	19.7 (5.7)	3 < 2 = 1 < No
**FAB**	16.0 (2.1)	13.2 (2.7)	12.1 (3.1)	9.5 (4.2)	3 < 2 = 1 < No

*Note.* Values indicate *M* (*SD*). CDR = Clinical Dementia Rating; NACC = National Alzheimer’s Coordinating Center; FTLD = frontotemporal lobar degeneration; MMSE = Mini-Mental State Examination; FAB = Frontal Assessment Battery.

a The CDR-weighted score was used for patients with AD, whereas the CDR^©^ plus NACC-FTLD-weighted score was used for patients with bvFTD, PPA, and controls.
